# Flavonoids Accumulation in Fruit Peel and Expression Profiling of Related Genes in Purple (*Passiflora edulis* f. *edulis*) and Yellow (*Passiflora edulis* f. *flavicarpa*) Passion Fruits

**DOI:** 10.3390/plants10112240

**Published:** 2021-10-20

**Authors:** Meng Shi, Muhammad Moaaz Ali, Yinying He, Songfeng Ma, Hafiz Muhammad Rizwan, Qiang Yang, Binqi Li, Zhimin Lin, Faxing Chen

**Affiliations:** 1 College of Horticulture, Fujian Agriculture and Forestry University, Fuzhou 350002, China; sm17720805309@163.com (M.S.); muhammadmoaazali@yahoo.com (M.M.A.); 18750357772@163.com (Y.H.); masongfeng2008@163.com (S.M.); chrizwan51@gmail.com (H.M.R.); yangqiang1720@163.com (Q.Y.); libinqi2020@126.com (B.L.); 2 Institute of Biotechnology, Fujian Academy of Agricultural Sciences, Fuzhou 350003, China

**Keywords:** *Passiflora edulis* Sims., *PAL*, fruit quality, *UFGT*, anthocyanin, UPLC-MS, qRT-PCR

## Abstract

Flavonoids play a key role as a secondary antioxidant defense system against different biotic and abiotic stresses, and also act as coloring compounds in various fruiting plants. In this study, fruit samples of purple (*Passiflora edulis* f. *edulis*) and yellow (*Passiflora edulis* f. *flavicarpa*) passion fruit were collected at five developmental stages (i.e., fruitlet, green, veraison, maturation, and ripening stage) from an orchard located at Nanping, Fujian, China. The contents of flavonoid, anthocyanin, proanthocyanin, and their metabolites were determined using ultra-performance liquid chromatography-mass spectrometry (UPLC-MS), activities of key enzymes involved in flavonoid metabolism were measured, and expression profiling of related genes was done using quantitative real-time PCR (qRT-PCR). The results revealed that total flavonoids, anthocyanins, and procyanidins were found to be increased in the fruit peel of both cultivars with fruit maturity. Total flavonoids, anthocyanins, procyanidins, flavonoid metabolites (i.e., rutin, luteolin, and quercetin), and anthocyanin metabolites (i.e., cyanidin-3-O-glucoside chloride, peonidin-3-O-glucoside, and pelargonidin-3-O-glucoside) were found abundant in the peel of purple passion fruit, as compared to yellow passion fruit. Principle component analysis showed that the enzymes, i.e., *C4H*, *4CL*, *UFGT,* and *GST* were maybe involved in the regulation of flavonoids metabolism in the peel of passion fruit cultivars. Meanwhile, *PePAL4, Pe4CL2,3, PeCHS2,* and *PeGST7* may play an important role in flavonoid metabolism in fruit peel of the passion fruit. This study provides new insights for future elucidation of key mechanisms regulating flavonoids biosynthesis in passion fruit.

## 1. Introduction

The passion fruit (*Passiflora edulis* Sims.) belongs to the Passifloraceae family, is native to tropical America, and has more than 500 species of which at least 50 or more are edible [1]. It is also called passion flower or egg fruit because it contains apple, guava, banana, strawberry, mango, pineapple, and 130 other kinds of fruit aroma [2,3]. The peel and pulp of passion fruit have many biological functions, such as controlling blood sugar [4], anti-hypertension [5,6], anti-inflammation and reducing fat [7], protecting liver and kidney [8], and regulating cardiac autonomic nerve functions [9]. In addition, passion fruit peel powder can be used as food raw material when added to baking products [10,11]. Passion fruit has high nutritional value and medicinal value and has great development potential [12].

Flavonoids widely exist in various horticultural plants and have a variety of biological activities [13], including chemoprophylaxis, inhibition of tumor growth [14], cancer prevention, anti-inflammation, and anti-oxidation [15], etc. In addition, flavonoids play an important role in preventing UV damage, signal transduction between plant and microorganisms, plant coloration, and defense [15]. The final flavonoid concentration in ripened fruits depends on the balance of flavonoid synthesis, membrane transport, and degradation or utilization [16,17]. In this process, flavonoid metabolism-related enzymes including L-phenylalanine ammonia-lyase (*PAL*), cinnamate 4-hydrogenase (*C4H*), 4-coumarate: coenzyme A Ligase (*4CL*), chalcone synthase (*CHS*), UPD-3-O- glycosyltransferase (*UFGT*), and glutathione S-transferase (*GST*) may potentially play a role in fruit flavonoids biosynthesis and degradation [18,19,20]. The phenylalanine pathway is an important pathway for the synthesis of many secondary metabolites [21]. *PAL* is the first key enzyme in this pathway, which catalyzes the decomposition of phenylalanine into cinnamic acid and enters the flavonoid synthesis pathway [22,23]. *4CL* is diversified by type III polyketone synthase (PKSs) to produce different products [24]. *CHS* is a key enzyme in the synthesis of naringin chalcone, which belongs to PKSs [25]. *UFGT*, as a key enzyme in anthocyanin synthesis, stabilizes anthocyanin mainly by attaching the sugar portion to anthocyanin glycogens [26]. *GST* plays a major role in anthocyanin transport, and the loss of function of these proteins results in the absence of a pigmentation phenotype in plants [27].

At present, *Passiflora edulis* f. *edulis* and *Passiflora edulis* f. *flavicarpa* are the main cultivated varieties, widely appreciated and accepted by consumers worldwide due to their unique flavor and high medicinal value [28,29]. There are obvious differences in the color of both cultivars during growth and development (especially at the veraison stage), which makes them good material to study the change in color of passion fruit peel [30]. Although the study of flavonoid metabolism pathway in plants has been very clear and in-depth and has been widely reported in fruits such as apples [31], mulberry [32], and grapes [33,34], dynamics of flavonoid contents, the activity of key enzymes encoding flavonoid synthesis and related gene expression during passion fruit development is still unclear. In this study, to explore the synthesis and accumulation mechanism of flavonoids and the regulation mechanism of structural genes in passion fruits, the content of flavonoid metabolites, components, activities of key enzymes related to flavonoid biosynthesis, and related structural genes in peel of purple and yellow passion fruits were analyzed at different developmental stages. It laid the foundation for revealing the mechanism of flavonoid metabolic pathway in passion fruit, functional analysis of related genes, research on bioactive substances, and development and utilization of fruit peel.

## 2. Results

### 2.1. Total Flavonoids, Anthocyanins, and Procyanidins

**Figure 1:**
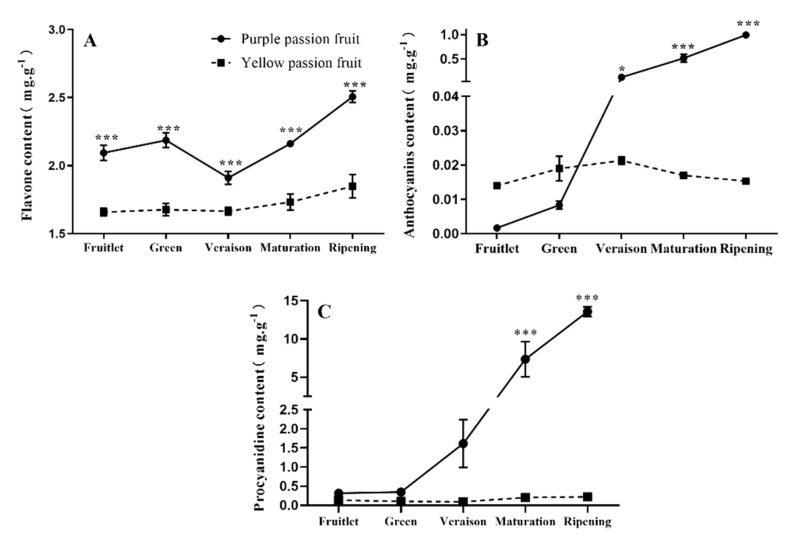
Changes in contents of total flavonoids (**A**), anthocyanin (**B**), and procyanidins (**C**) in the peel of purple and yellow passion fruit during fruit development. Vertical bars indicate means ± SD (*n* = 3, 5 fruits per replicate). The * and *** represent significance at *p* ≤ 0.05 and *p *≤ 0.001, respectively, among both cultivars according to Student’s *t*-test.

The total flavonoid content in the peel of purple passion fruit was significantly higher than that in yellow passion fruit (Figure 1A). With the development and maturity of passion fruit, the changing trend of flavonoids in purple passion fruit and yellow fruit peel showed an inverted “L” pattern, first decreasing and then increasing. The maximum flavonoids (2.5 mg·g^−1^) were recorded in purple passion fruit at the ripening stage.

Anthocyanin content in the peel of purple passion fruit increased with increase in fruit maturity, while in yellow passion fruit, it increased till the veraison stage and then decreased. The anthocyanin content of purple passion fruit peel was significantly higher from veraison to the ripening stage than that of other stages and reached 1.0 mg·g^−1^ at late ripening stage (Figure 1B). In purple passion fruit, procyanidin content increased gradually with fruit development, while this profile was scarcely present in yellow passion fruits at all studied stages. The maximum procyanidin (13.58 mg·g^−1^) was recorded in the peel of purple passion fruit at ripening stage (Figure 1C).

### 2.2. Flavonoid and Anthocyanin Metabolites

Five flavonoid (i.e., rutin, luteolin, quercetin, apigenin, and kaempferol) and three anthocyanin metabolites (cyanidin-3-O-glucoside chloride, peonidin-3-O-glucoside, and pelargonidin-3-O-glucoside) were determined in the peel of purple and yellow passion fruits. Apigenin and kaempferol were not detected in all fruit samples, and the other three flavonoids were detected in the fruit peel of both cultivars.

The content of flavonoid components measured in purple passion fruit was significantly higher than that in yellow passion fruit. In purple passion fruit peel, the contents of six components increased gradually with fruit development, besides luteolin and quercetin which decreased slightly at the green fruit stage. The contents of rutin, luteolin, and quercetin reached the highest level at the ripening stage in purple passion fruit, 22,569.60, 29.19, and 35.25 ng·g^−1^, respectively. Similarly, the cyanidin-3-O-glucoside chloride, peonidin-3-O-glucoside, and pelargonidin-3-O-glucoside contents were also the highest in purple passion fruit at the ripening stage, which were 7341.62 ng·g^−1^, 9793.08 ng·g^−1^, and 511.92 ng·g^−1^, respectively. In the yellow passion fruit, the contents of rutin and luteolin decreased at veraison and then increased at the ripening stage. Quercetin continued to decrease with fruit development and was not detected at the ripening stage. The contents of anthocyanin components in yellow passion fruit were far less, as compared with the purple passion fruit (Table 1).

### 2.3. Key Enzymes Involved in Flavonoids Metabolism

**Figure 2:**
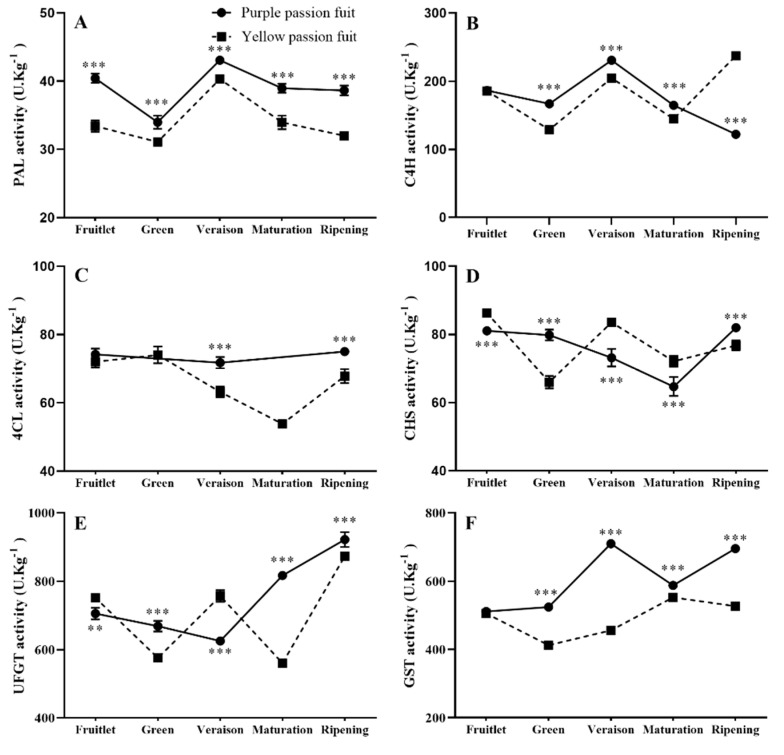
Changes in *PAL* (**A**), *C4H* (**B**), *4CL* (**C**), *CHS* (**D**), *UFGT* (**E**)*,* and *GST* (**F**) activities of purple and yellow passion fruit peel during fruit growth and development. Vertical bars indicate means ± SD (*n* = 3, 5 fruits per replicate). The **, and *** represent significance at *p* ≤ 0.01, and *p* ≤ 0.001, respectively, among both cultivars according to Student’s *t*-test.

The *PAL* activity in the peel of both cultivars reached its maximum level (43.31 U·Kg^−1^) at the veraison stage, and then gradually decreased with fruit maturity. The changing trend of *PAL* activity in purple passion fruit was significantly higher than that of yellow passion fruit at each stage (Figure 2A). There were significant differences in the *C4H* enzyme activity of both cultivars at each stage, except for the fruitlet stage. The maximum *C4H* enzyme activity was recorded in purple (232.10 U·Kg^−1^) and yellow passion fruit (236.34 U·Kg^−1^) at the veraison and ripening stage, respectively (Figure 2B). The *4CL* activity in purple passion fruit was stable, first decreasing and then increasing slightly during fruit development, and there was no significant difference between fruitlet and maturation stage. In yellow passion fruit, *4CL* activity decreased at the maturation stage to the lowest level (53.62 U·Kg^−1^) and increased at the ripening stage. After the veraison stage, the *4CL* activity of purple passion fruit was significantly (*p ≤ 0.001)* higher than that of yellow passion fruit (Figure 2C).

The *CHS* activity of the peel of both examined passion fruit cultivars ranged from 65.17 to 86.77 U·Kg^−1^. The maximum activity was recorded at the green stage of yellow passion fruit. The *CHS* activity in purple and yellow passion fruit was significantly different, and the variation trend was also different (Figure 2D).

The changes in *UFGT* activity in the peel of both cultivars were significantly different at different fruit development stages. In purple passion fruit, the *UFGT* activity decreased at the veraison stage and increased sharply at the maturation and ripening stages. The difference between purple and yellow fruit passion fruit reached the maximum level at the maturation stage, and purple passion fruit was about 250 U·Kg^−1^ higher than yellow passion fruit (Figure 2E).

There were significant differences in *GST* activity between purple and yellow passion fruit peel, especially at the veraison and ripening stage. It reached the maximum in purple passion fruit, which was about 1/3 times higher than that in yellow passion fruit (Figure 2F).

### 2.4. Expression Profiling of Genes Encoding Key Enzymes for Flavonoid Metabolism

**Figure 3:**
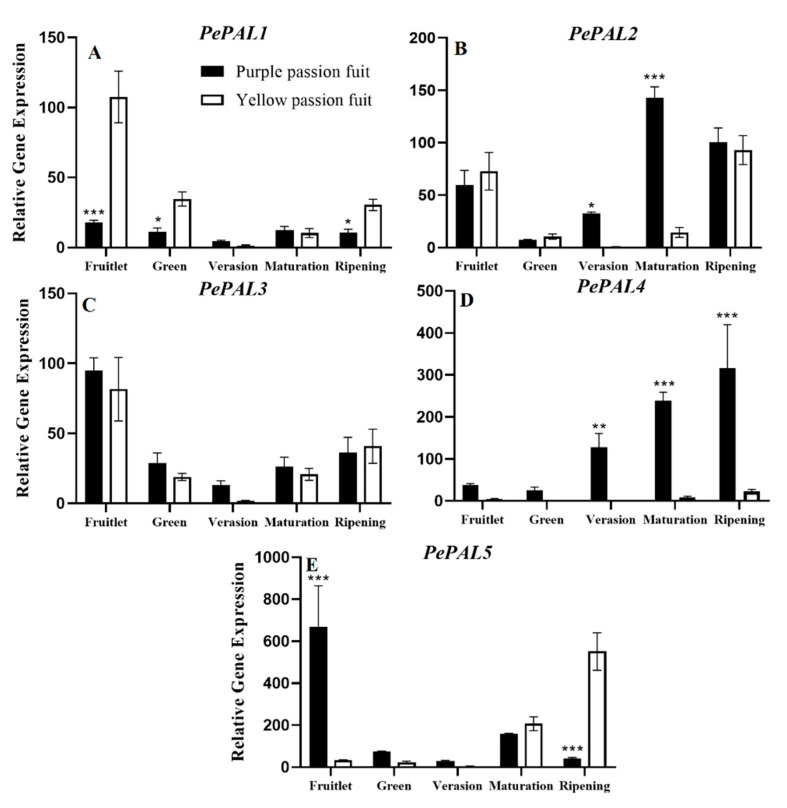
Relative expressions of *PePAL1-5* (**A**–**E**) genes in purple and yellow passion fruit peel during fruit growth and development. The relative gene expression was calculated using the 2^−ΔΔct^ method. Vertical bars indicate means ± SD (*n* = 3, 5 fruits per replicate). The *, **, and *** represent significance at *p* ≤ 0.05, *p* ≤ 0.01, and *p* ≤ 0.001, respectively, among both cultivars according to Student’s *t*-test.

The core genes i.e., *PePAL1–5*, *PeC4H*, *Pe4CL1–7*, *PeCHS1–3*, *PeUFGT1–2*, and *PeGST1–7* responsible for the biosynthesis of *PAL*, *C4H*, *4CL*, *CHS*, *UFGT,* and *GST* were studied, respectively. During passion fruit development, the relative expressions of five *PePAL* genes in both passion fruit cultivars decreased first and then increased, and showed a down-regulated trend before the veraison stage, while it was up-regulated after the veraison stage. The relative expression levels of *PePAL2* and *PePAL4* in purple passion fruit peel were significantly (*p ≤ 0.001)* higher than that in yellow passion fruit after the veraison stage. Interestingly, at the ripening stage, purple passion fruit exhibited 14.34-fold more genetic expression of *PePAL4* as compared to that of yellow passion fruit. Conversely, the relative expression of *PePAL5* was 13.79-times higher in yellow passion fruit compared with purple passion fruit at the fruit ripening stage (Figure 3).

**Figure 4:**
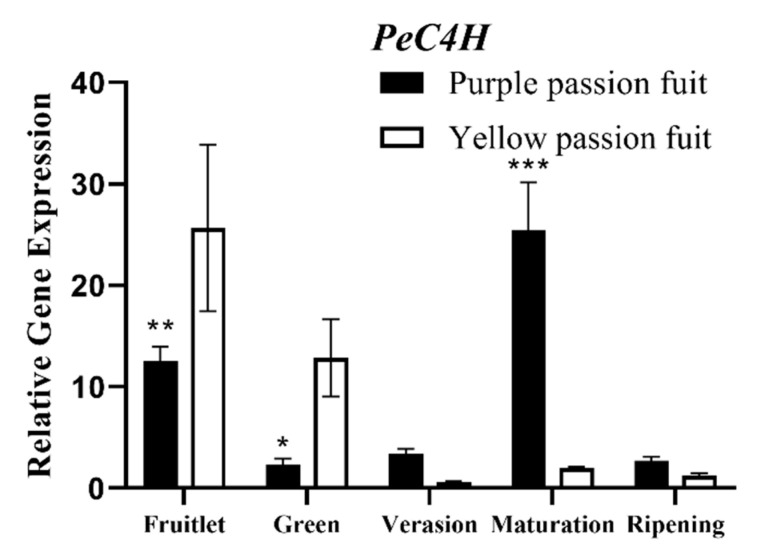
Relative expressions of *PeC4H* gene in purple and yellow passion fruit peel during fruit growth and development. The relative gene expression was calculated using the 2^−ΔΔct^ method. Vertical bars indicate means ± SD (*n* = 3, 5 fruits per replicate). The *, **, and *** represent significance at *p* ≤ 0.05, *p* ≤ 0.01, and *p* ≤ 0.001, respectively, among both cultivars according to Student’s *t*-test.

*PeC4H1* gene was significantly expressed in fruit peel, and the expression level of *PeC4H1* gene was higher in yellow passion fruit before the veraison stage, but significantly increased in purple fruit after the veraison stage, and was higher than that in yellow passion fruit (Figure 4).

**Figure 5:**
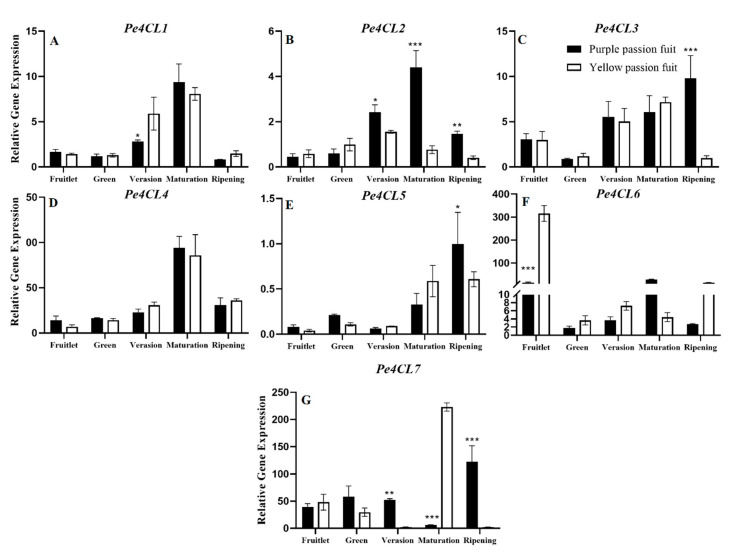
Relative expressions of *Pe4CL1-7* (**A**–**G**) genes in purple and yellow passion fruit peel during fruit growth and development. The relative gene expression was calculated using the 2^−ΔΔct^ method. Vertical bars indicate means ± SD (*n* = 3, 5 fruits per replicate). The *, **, and *** represent significance at *p* ≤ 0.05, *p* ≤ 0.01, and *p* ≤ 0.001, respectively, among both cultivars according to Student’s *t*-test.

The expression patterns of *Pe4CL1, Pe4CL2,* and *Pe4CL4* showed a parabolic pattern, *Pe4CL3* and *Pe4CL5* showed ‘J’ pattern, and *Pe4CL6* and *Pe4CL7* varied during fruit development. *Pe4CL2, Pe4CL3,* and *Pe4CL7* were significantly expressed in fruit peel after the veraison stage. Both examined cultivars showed non-significant different in terms of *Pe4CL1* and *Pe4CL4* expression patterns (Figure 5A,D). *Pe4CL2* was significantly (*p* ≤ 0.001) expressed in purple passion at the maturation stage as compared to yellow passion fruit, showing its highest expression (4.41) (Figure 5B). The maximum expression of *Pe4CL3* (9.80) was observed in the peel of purple passion fruit at the fruit ripening stage, which was 10-fold higher than that in yellow passion fruit (Figure 5C). Similarly, purple passion fruit exhibited 1.64-fold higher expression of *Pe4CL5* at the ripening stage as compared to yellow passion fruit (*p* ≤ 0.05) (Figure 5E). The relative expression of *Pe4CL6* and *Pe4CL7* varied during fruit development of both passion fruit cultivars (Figure 5F,G).

**Figure 6:**
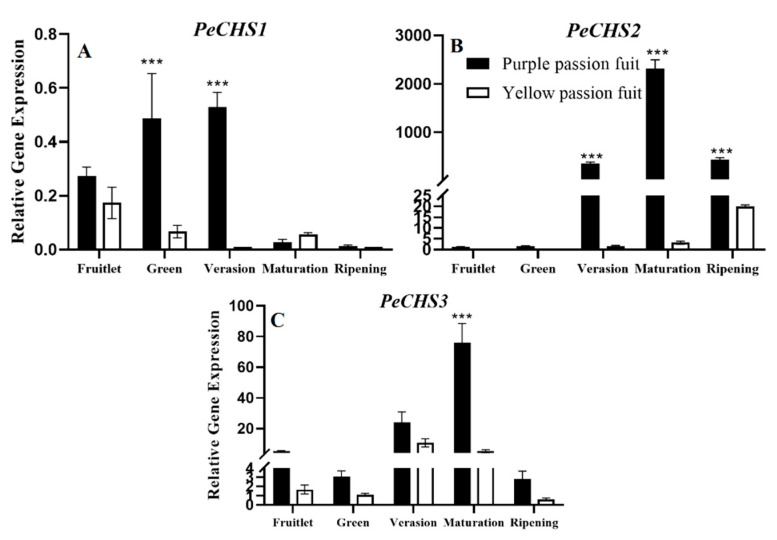
Relative expressions of *PeCHS1-3* (**A**–**C**) genes in purple and yellow passion fruit peel during fruit growth and development. The relative gene expression was calculated using the 2^−ΔΔct^ method. Vertical bars indicate means ± SD (*n* = 3, 5 fruits per replicate). The *** represents significance at *p* ≤ 0.001, among both cultivars according to Student’s *t*-test.

Purple passion fruit showed overall more expression of *PeCHS* gene in fruit peel compared with yellow passion fruit. The expression of *PeCHS1* was the highest (0.53) in purple passion fruit at the veraison stage, which was 48-times higher than that in yellow passion fruit (Figure 6A). The expression levels of *PeCHS2* and *PECHS3* were highest in purple passion fruit at maturation stage (2309.05 and 75.94, respectively) (Figure 6B,C).

**Figure 7:**
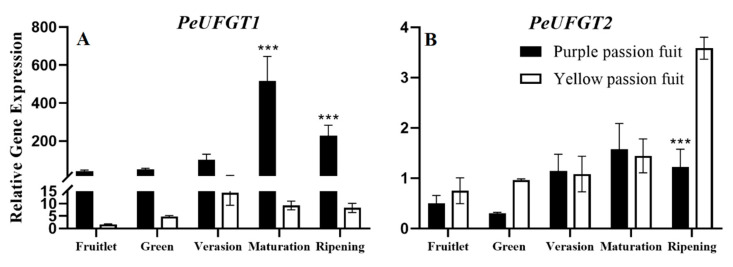
Relative expressions of *PeUFGT1* (**A**) and *PeUFGT2* (**B**) genes in purple and yellow passion fruit peel during fruit growth and development. The relative gene expression was calculated using the 2^−ΔΔct^ method. Vertical bars indicate means ± SD (*n* = 3, 5 fruits per replicate). The *** represents significance at *p* ≤ 0.001, among both cultivars according to Student’s *t*-test.

The expression of *PeUFGT1* in purple passion fruit was significantly higher than that in yellow passion fruit, which observed a maximum (516.03) at the maturation stage. In terms of *PeUFGT2* expression, there was a non-significant difference among both cultivars before the ripening stage, while at the ripening stage yellow passion fruit exhibited 2.94-fold higher expression than that in purple passion fruit (Figure 7).

**Figure 8:**
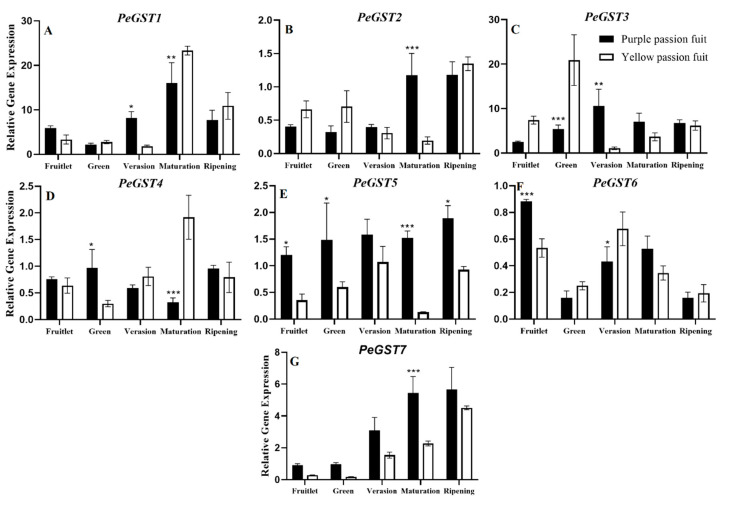
Relative expressions of *PeGST1-7* (**A**–**G**) genes in purple and yellow passion fruit peel during fruit growth and development. The relative gene expression was calculated using the 2^−ΔΔct^ method. Vertical bars indicate means ± SD (*n* = 3, 5 fruits per replicate). The *, **, and *** represent significance at *p* ≤ 0.05, *p* ≤ 0.01, and *p* ≤ 0.001, respectively, among both cultivars according to Student’s *t*-test.

The expressions of *PeGST1* and *PeGST2* was significantly different among purple and yellow passion fruit peel at the maturation stage. Yellow passion fruit showed more expression level of *PeGST1* (23.33), while purple passion fruit exhibited more *PeGST2* expression (1.18) at maturation stage (Figure 8A,B). The expressions of *PeGST3* and *PeGST4* were higher in yellow passion fruit as compared to purple passion fruit at the green and maturation stage, respectively (Figure 8C,D). The expression levels of *PeGST5* and *PeGST7* in purple passion fruit were generally higher than those in yellow passion fruit during development, while the other *PeGST* expressions were different in individual development stages (Figure 8E,G). *PeGST6* expression was significantly different (*p* ≤ 0.001) among both passion fruit cultivars at fruitlet stage, while showed varied pattern at other maturity stages (Figure 8F).

### 2.5. Principle Component Analysis

#### 2.5.1. Purple Passion Fruit

**Figure 9:**
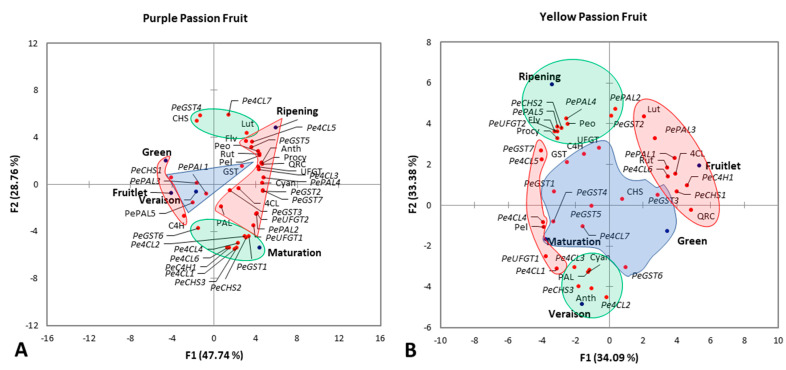
Principal component analysis among total flavonoids, anthocyanins, procyanidins, flavonoid and anthocyanin metabolites, key enzymes, and relative expressions of genes involved in flavonoids metabolism of purple (**A**) and yellow (**B**) passion fruit. Clustering of fruit maturity stages and measured attributes into groups (colored shapes) is based on their highest squared cosine values corresponding to the factor, F1 (red), F2 (green) or F3 (blue). Abbreviations: *Flv*—total flavonoids; *Anth*—total anthocyanins; *Procy*—total procyanidins; *Rut*—rutin; *Lut*—luteolin; *QRC*—quercetin; *Cyan*—cyanidin-3-O-glucoside chloride; *Peo*—peonidin-3-O-glucoside; *Pel*—pelargonidin-3-O-glucoside; *PAL*—L-phenylalanine ammonia-lyase; *C4H*—cinnamate 4-hydrogenase; *4CL*—4-coumarate: coenzyme A Ligase; *CHS*—chalcone synthase; *UFGT*—UPD-3-O- glycosyltransferase; *GST*—glutathione S-transferase.

Principal component analysis (PCA) was conducted to assess the correlation between fruit maturity stages and flavonoid attributes of passion fruit. Based on the highest squared cosine values corresponding to PCA factors (F1, F2 or F3), flavonoids, anthocyanins, procyanidins, flavonoid and anthocyanin metabolites, key enzymes, and relative expressions of genes involved in flavonoids metabolism were clustered around the maturity stages of passion fruit. Factor F1, covering 47.74% variability in data (eigenvalue 19.096), showed clustering of total flavonoids, anthocyanins, procyanidins, rutin, quercetin, cyanidin-3-O-glucoside chloride, peonidin-3-O-glucoside, pelargonidin-3-O-glucoside, UFGT, *PePAL2*, *PePAL4*, *Pe4CL3*, *Pe4CL5*, *PeCHS1*, *PeUFGT1*, *PeUFGT2*, *PeGST2*, *PeGST5*, and *PeGST7* with ripening stage of purple passion fruit, suggesting positive correlation of these variable among each other. C4H was significantly correlated with fruitlet and green fruiting stage of passion fruit. This cluster was located opposite to ripening on the F1 axis, suggesting strong negative association of the ripening stage with C4H. The second factor, covering 28.76% variability in data (eigenvalue 11.504), showed clustering of luteolin, CHS, *PeC4H1*, *Pe4CL1*, *Pe4CL2*, *Pe4CL4*, *Pe4CL6*, *Pe4CL7*, *PeCHS2*, *PeCHS3*, *PeGST1*, *PeGST4*, and *PeGST6* with the maturation stage of purple passion fruit. However, the distribution of clusters in two distinct groups on either side of the F2 axis indicated that the maturation stage had positive correlation with *PeC4H1*, *Pe4CL1*, *Pe4CL2*, *Pe4CL4*, *Pe4CL6*, *PeCHS2*, *PeCHS3*, *PeGST1*, and *PeGST6* but negative correlation with luteolin, CHS, *Pe4CL7*, and *PeGST4*. The third factor of PCA, covering 15.92% variability in data (eigenvalue 6.370), showed clustering of GST, *PePAL1*, *PePAL3*, *PePAL5*, and *PeGST3* with the veraison stage (Figure 9A).

#### 2.5.2. Yellow Passion Fruit

Factor F1 of PCA, covering 34.09% variability in data (eigenvalue 13.636), showed clustering of rutin, quercetin, pelargonidin-3-O-glucoside, 4CL, *PePAL1*, *PeC4H1*, *Pe4CL1*, *Pe4CL4*, *Pe4CL5*, *Pe4CL6*, *PeCHS1*, *PeUFGT1*, and *PeGST7* with the fruitlet stage of yellow passion fruit, suggesting positive correlation of these variables among each other. The distribution of clusters in two different groups on either side of F1 axis indicated that the fruitlet stage had positive correlation with rutin, quercetin, 4CL, *PePAL1*, *PeC4H1*, *Pe4CL1*, *Pe4CL6*, and *PeCHS1* but negative correlation with pelargonidin-3-O-glucoside, *Pe4CL4*, *Pe4CL5*, *PeUFGT1*, and *PeGST7*. The second factor, covering 33.38% variability in data (eigenvalue 13.350), showed clustering of anthocyanins, cyanidin-3-O-glucoside chloride, PAL, *PePAL3*, *Pe4CL2*, *Pe4CL3*, and *PeCHS3* with the veraison stage of yellow passion fruit. Total flavonoids, procyanidins, peonidin-3-O-glucoside, *PePAL2*, *PePAL4*, *PePAL5*, *PeCHS2*, *PeUFGT2*, and *PeGST2* showed positive correlation with ripening stage. The third factor of PCA, covering 18.69% variability in data (eigenvalue 7.476), showed clustering of luteolin, UFGT, C4H, CHS, GST, *Pe4CL7*, *PeGST1*, *PeGST3*, *PeGST4*, *PeGST5*, and *PeGST6* with the maturation stage of yellow passion fruit (Figure 9B).

## 3. Discussion

Flavonoids are a widely distributed group of phenolics, occurring virtually in all plant parts. They are a major coloring substance in flowers and fruits. They also play a vital role as a secondary antioxidant defense system against different biotic and abiotic stresses [35]. Flavonoids are located within the centers of ROS generation and in the nucleus of mesophyll cells [36]. Flavonoid components have been reported in the leaves [37], fruit peel [38], and pulp [39] of passion fruit. In current study, the contents of flavonoids, anthocyanins, and procyanidins in fruit peel of purple and yellow passion fruit showed great differences. During fruit growth and development, the contents of flavonoids, anthocyanins, and procyanidins in purple passion fruit peel were significantly higher than that of yellow passion fruit, and the difference reached the maximum at ripening stage (Figure 1).

Five flavonoid (i.e., rutin, luteolin, quercetin, apigenin, and kaempferol) and three anthocyanin components (cyanidin-3-O-glucoside chloride, peonidin-3-O-glucoside, and pelargonidin-3-O-glucoside) were determined by UPLC-MS in the peel of purple and yellow passion fruits (Table 1). Apigenin and kaempferol were almost not detected in all fruit samples (Table S1), but could be detected in passion fruit leaves (unpublished data). Ferreres et al. [37] detected a variety of apigenin substances in the study on the antioxidant activity of passion fruit leaves. During fruit development, flavonoid and anthocyanin components detected in the peel of purple passion fruit were significantly higher than that of yellow passion fruit. Rutin content was the highest among all detected flavonoids, which was consistent with many earlier findings [40,41]. The cyanidin-3-O-glucoside chloride has been considered as the quantitative standard of anthocyanins in many crops [37,42,43], while in the current study, peonidin-3-glucoside was found maximum in the peel of purple and yellow passion fruit among measured anthocyanin components at the ripening stage (Table 1). Luteolin was detected in abundance at earlier stages of fruit ripening in the peel of the fruits of both cultivars. A rare passion fruit variety (*Passiflora loefgrenii* Vitta.) studied by Argentieri et al. [44] is rich in luteolin, making it a good choice for biopharmaceuticals. The luteolin content at the ripening stage of the purple passion fruit was much higher than that in other growth stages, which can be further studied or used as an important growth stage for the extraction of luteolin.

**Figure 10:**
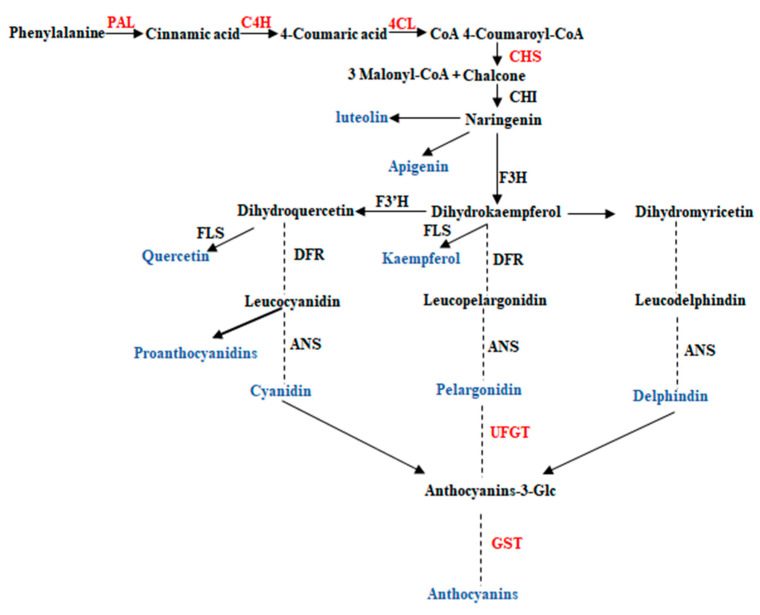
Flavonoids’ biosynthesis pathway in plants.

Flavonols are converted to anthocyanins and other flavonoid substances through the catalysis of different enzymes [45] (Figure 10). Phenylalanine is the direct precursor of flavonoid synthesis, and the first stage is the conversion of phenylalanine to 4-coumaryl CoA. *PAL*, *C4H*, and *4CL* are the main regulatory enzymes involved in this process [46]. The second stage is the conversion of 4-coumaryl CoA and 3 malonyl CoA to dihydroxyflavonol, which is the key reaction of flavonoid metabolism. *CHS*, *CHI* and *F3H* activity regulate this reaction [47]. The third stage is the synthesis of unmodified anthocyanins [48]. Finally, it is modified by glycosyltransferase (*GT*) and transported to vacuole by *GST* [49].

The changes in enzymatic activities fruit growth and development of passion fruit are complicated. The variation trend of *PAL* activity in both passion fruit cultivars was similar, but the magnitude was significantly different (Figure 2A). *PAL* activity has been reported different in two rapeseed (*Brassica napus*) cultivars, and due to difference in *PAL* activity, different biological activities regulated different metabolic pathways [50]. The changing trend and the activity level of other measured enzymes were also different in the peel of both passion fruit cultivars (Figure 2). The *PAL*, *CHS*, *C4H,* and *4CL* genes have important regulatory effects on flavonoid synthesis in plants [51]. The RT-qPCR results of the structural genes corresponding to the six flavonoid metabolism-related enzymes showed that they had different expression patterns in the fruit peel (Figure 3, Figure 4, Figure 5, Figure 6, Figure 7 and Figure 8) of the purple and yellow passion fruit. The relative expression levels of *PePAL2*,*4*, *PeC4H1*, *Pe4CL2*,*3*,*7*, *PECHS1–3*, *PeUFGT1,* and *PeGST5*,*7* genes in purple passion fruit were significantly (*p* ≤ 0.05) higher than those in yellow passion fruit during peel development. The difference of flavonoid metabolites in passion fruit is related to the activity of these enzymes and the differential expression of corresponding structural genes at different developmental stages, and the specific mode of action needs to be further verified. There are seven main metabolic pathways of flavonoids in plants, i.e., anthocyanins pathway, proanthocyanidins (condensed tannins) pathway, flavones pathway, flavanols pathway, isoflavone pathway, tanning anhydride pathway, and Aurones pathway [52]. This study mainly studied the initial stages of the synthesis pathway from phenylalanine to anthocyanin (Figure 10). The key genes i.e., *PAL, C4H, 4CL, CHS* were involved in the biosynthesis of phenylalanine to chalcone in pathway, and *UFGT* and *GST* played a role in the glycosylation and transport of anthocyanins.

In this study, PCA showed that flavonoid metabolites in the peel of purple passion fruit were negatively correlated with *C4H* and positively correlated with *4CL*, *UFGT,* and *GST* enzyme activities. Interestingly, *C4H* and *UFGT* enzyme activities showed a significant positive correlation with each other in yellow passion fruit peel, while they showed a negative correlation in purple fruit peel. Therefore, these two enzymes are likely to be the key enzymes affecting the metabolism of flavonoids in purple and yellow passion fruit peel (Figure 9). *PAL* and *C4H* were positively correlated with flavonoid content in tobacco [53], while *CHS* expression was significantly positively correlated with anthocyanin content in pomegranate [54]. Flavonoid related synthetic genes showed early and late expression peaks in grape [55], *Vaccinium myrtillus* [49] and wild apple (*Malus Sylvestris* L.) [56]. The PCA revealed that *PePAL4, Pe4CL2,3, PeCHS2,* and *PeGST7* may play an important role in flavonoid metabolism in fruit peel of passion fruit. The differential accumulation of flavonoid-related metabolites in both passion fruit cultivars was not only related to enzymatic activity but also structural gene expressions. According to previous studies, it was also related to some transcription factors such as *MYB* [57], *BHLH* [58], and *WD40* [59], regulation of microRNA during protein expression and, ubiquitination and phosphorylation during protein activation [60].

## 4. Materials and Methods

### 4.1. Plant Material

**Figure 11:**
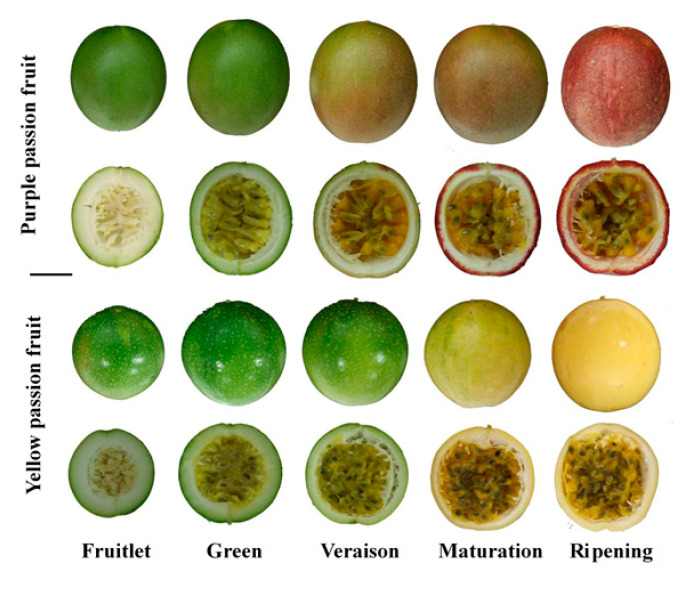
Lateral and transverse sections of fruits at different developmental stages of purple and yellow passion fruits.

The plant material of two passion fruit cultivars, i.e., purple passion fruit (Tainong No. 1) and yellow passion fruit (Golden) was obtained from a passion fruit orchard, located at Shaowu county, Nanping city, Fujian province, China (27°22′51.9″ N 117°32′18.4″ E). The 15 passion fruits from each cultivar were sampled at each developmental stage, i.e., fruitlet, green, veraison, maturation, and repining (Figure 11). After shifting the fruits to the laboratory (Institute of Tropical and Subtropical Fruit Trees, College of Horticulture, Fujian Agriculture and Forestry University, China), the peel (separated sponge layer) of five passion fruits were mixed as one biological replicate, with three biological replicates per sample. All samples were stored in −80 °C ultra-low temperature refrigerator for later use.

### 4.2. Determination of Total Flavonoids, Anthocyanins, and Proanthocyanins

Extraction of total flavonoids was based on the optimization method of Vinatoru et al. [61] with some modifications. Accurately weighed 0.2 g of −80 °C frozen fruit peel was added to 8 mL of 60% ethanol. The solution was subjected to ultrasonic extraction for 40 min, cooling for 20 min, and centrifugation for 10 min (8000 rpm, 20 °C). The 5 mL supernatant was taken and diluted with distilled water to make the final volume of 10 mL. After that, 2 mL aliquot was taken and 3 mL of 60% ethanol and 0.3 mL of 5% NaNO_2_ were added. After shaking well and waiting for 6 min, 0.3 mL of 10% Al(NO_3_)_3_ was added. After shaking and then resting for 6 min, 4 mL of 4% NaOH was added. After shaking well and resting for 12 min, absorbance was measured at 510 nm. Total flavonoid content was calculated using calibration curve (Y = 10.859X − 0.0617, R^2^ = 0.999) of rutin standard (HPLC grade, ≥98% purity, Solarbio Life Sciences, Beijing, China).

Total anthocyanins were extracted following the protocol earlier described by Kim and Lee [62]. The 0.2 g plant material was added into 10 mL of 1% hydrochloric acid methanol solution and kept for 5 h. After centrifugation at 1000 rpm for 20 min, 10 mL supernatant was used to measure the OD value of the sample at 530 nm and 560 nm. Equation (1) was used to calculate total anthocyanins.

(1) $$\mathrm{Total}\;\mathrm{anthocyanins}\;(\mathrm{mg}\cdot g^{-1})=\frac{\left(\mathrm{OD}530-0.25\times \mathrm{OD}650\right)\times \;\mathrm{volume}\;\mathrm{of}\;\mathrm{extraction}\;\mathrm{liquid}\;\left(\mathrm{mL}\right)}{4.62\times 104\times \;\mathrm{fresh}\;\mathrm{weight}\;\mathrm{of}\;\mathrm{passion}\;\mathrm{fruit}\;\left(g\right)}\;.$$

Procyanidin content was determined using the method of Hellstrom and Mattila [63], with slight modifications. The 0.5 g sample was accurately weighed in a 10 mL centrifuge tube, 6 mL methanol was added, and ultrasonic treatment (power = 250, yield = 50 kHz) was conducted for 20 min. After being placed at room temperature, the supernatant was centrifuged to measure the absorbance at 546 nm. The procyanidin content was calculated using calibration curve (Y = 0.0038X + 0.0202, R^2^ = 0.999) of procyanidin standard (HPLC grade, ≥95% purity, Solarbio Life Sciences, Beijing, China).

### 4.3. Determination of Flavonoid and Anthocyanin Metabolites

For sample preparation, the method earlier described by Henry-Kirk et al. [64] was used with some changes. The 1 g plant material was ground along with liquid nitrogen, and 5 mL of methanol/formic acid/water (80:1:19, *v/v/v*) was added. Ultrasonic extraction was performed at 45 °C for 60 min, and centrifugation was performed at 12,000 rpm for 10 min, and the supernatant was filtered through MFMillipore™ Membrane Filter (Cat. No. GSWP04700, 0.22 µm pore size) into an Agilent sample bottle for testing. Five standard flavonoids of rutin, quercetin, luteolin, apigenin, and kaempferol (≥98% purity, Solarbio Life Sciences, Beijing, China) were prepared with the concentration of 0.1 mg·mL^−1^, and three standard anthocyanins of cyanidin-3-O-glucoside chloride (≥98% purity, Solarbio Life Sciences, Beijing, China), peonidin-3-O-glucoside (≥95% purity, Solarbio Life Sciences, Beijing, China), pelargonidin-3-O-glucoside (≥95% purity, Solarbio Life Sciences, Beijing, China) were prepared. Ultra-performance liquid chromatography-mass spectrometry (UPLC-MS) was performed with Waters I-CLASS /XEVO TQS liquid mass spectrometer (Waters Corporation, Milford, MA, USA) for evaluation. The determination was performed on the Agilent-ZORBAX SB-C18 column at the flow rate of 0.3 mL·min^−1^ and the column temperature was 40 °C. The flavonoid and anthocyanin components were detected at 210 nm. A Waters 2996 diode array detector (Waters Corporation, Milford, MA, USA) was used to detect the eluted peaks. The contents of individual flavonoid or anthocyanin metabolites were calculated using calibration curve of the corresponding standard. All measurements were performed with three replicates. The validation parameters consisted of linearity range, limits of detection, and quantification [65]. The peaks were identified by their retention times, comparing the UV–Visible spectra and spiking with standards. Quantification has been done using an external standard curve with five points (Table 2).

### 4.4. Enzymes Extraction and Activity Assay

Flavonoid metabolism-related enzymes including L-phenylalanine ammonia-lyase (*PAL*), cinnamate 4-hydrogenase (*C4H*), 4-coumarate: coenzyme A Ligase (*4CL*), chalcone synthase (*CHS*), UPD-3-O- glycosyltransferase (*UFGT*), and glutathione S-transferase (*GST*) were extracted and measured using the Solarbio enzyme activity kits (Solarbio Life Sciences, Beijing, China) according to the manufacturer’s instructions [66,67].

### 4.5. RNA Extraction and Real-Time Quantitative PCR

Based on transcriptome data of passion fruit at different developmental stages, differential candidate sequences of *PAL, C4H, 4CL, CHS, UFGT,* and *GST* were identified by KEGG metabolic pathway analysis of phenylalanine, flavonoids, and isoflavones enriched in passion fruit. Local BLAST screening of homologous genes was performed by BioEdit software (v 7.2). Then, the preliminarily obtained genes were put into NCBI for BLAST comparison and SMART (http://smart.embl-heidelberg.de/, accessed on 16 November 2020) conserved domain analysis to screen out the preliminary candidate genes. The genes were compared with those from the published passion fruit genome (http://ftp.cngb.org/pub/CNSA/data3/CNP0001287/CNS0275691/CNA0017758/, accessed on 16 November 2020). According to the Unigenes sequence in the transcriptome, qRT-PCR specific primers were designed using Primer 5 online software [68] (Table S2). TIANGEN polysaccharide polyphenol plant TOTAL RNA extraction kit (centrifugal column) was used to extract total RNA from yellow and purple passion fruit at different developmental stages in strict accordance with the instructions. The first strand of cDNA was synthesized using TaKaRa’s quantitative reverse transcription kit, and fluorescence quantitative PCR was performed using LightCycler^®^ 96 quantitative instrument (Roche Applied Science, Penzberg, Germany).

The reaction mixture contained 10 µL 2 × RealStar Green Fast Mixture (GenStar, Bejing, China), 1 µL cDNA, 0.25 µM of each primer, and water was added to make a final volume of 20 µL. Cycling conditions were as follows: 95 °C for 2 min, 40 cycles of 95 °C for 5 s, and 60 °C for 30 s. The 60 S ribosomal protein was used as an internal control, and the relative gene expression was calculated using the 2^−ΔΔct^ method [69]. Three independent biological replicates were analyzed for each sample.

### 4.6. Statistical Data Analysis

Collected data at each fruit maturity stage were subjected to one-way analysis of variance (ANOVA) using GraphPad Prism 8.0.1 (https://www.graphpad.com/scientific-software/prism/, accessed on 21 June 2021). Comparison between ‘yellow’ and ‘purple’ passion fruit for each developmental stage was performed using Student’s *t*-test. Flavonoid metabolites of each cultivar were compared between different developmental stages using Fisher’s least significant difference technique through analytical software package “SPSS statistics 21.0” (IBM Inc., New York, NY, USA). Principle component analysis and correlation coefficient values were determined with Pearson (*n*) method using the XLSTAT ver. 2019.

## 5. Conclusions

In this study, the flavonoids biosynthesis mechanism of two passion fruit cultivars having fruits of different color (purple and yellow) was studied. The content of flavonoid components and metabolites, activities of key enzymes related to its biosynthesis, and expressions of flavonoids-related structural genes in fruit peel of both passion fruit cultivars were analyzed at different developmental stages. The results revealed that the maximum content of flavonoid metabolites was observed in the peel of purple passion fruit. The dynamics of the flavonoid contents measured in the current study were not solely controlled by a single enzyme but were regulated by the integrated activity of different enzymes such as *PAL*, *C4H*, *4CL*, *CHS*, *UFGT,* and *GST*. Among them, *C4H*, *4CL*, *UFGT,* and *GST* played a significant role in flavonoids accumulation in passion fruit peel. *PePAL4, Pe4CL2,3, PeCHS2,* and *PeGST7* had a great influence on the metabolism of flavonoids in fruit peel. These results provided new insight into the characteristics of flavonoids metabolism and are a valuable resource for future research on molecular breeding in passion fruit.

## Figures and Tables

**Table 1:** The content of flavonoid and anthocyanin metabolites in fruit peel of purple and yellow passion fruits during fruit development.

Cultivar	Fruiting Stage	Rutin (ng/g)	Luteolin (ng/g)	Quercetin (ng/g)	Cyanidin-3-O-Glucoside Chloride (ng/g)	Peonidin-3-O-Glucoside (ng/g)	Pelargonidin-3-O-Glucoside (ng/g)
	Fruitlet	906.99 ± 79.10 c	15.62 ± 0.48 b	5.55 ± 2.31 c	1.48 ± 0.58 d	1.13 ± 0.85 c	3.53 ± 0.53 c
	Green	1386.36 ± 132.25 c	13.55 ± 0.88 b	4.63 ± 4.50 c	3.91 ± 0.60 d	2.02 ± 0.19 c	4.15 ± 0.39 c
Purple	Veraison	2384.89 ± 1309.13 c	13.50 ± 3.05 b	9.01 ± 5.30 c	3881.42 ± 572.51 c	411.03 ± 310.05 c	75.94 ± 32.73 c
	Maturation	9499.92 ± 692.22 b	13.91 ± 1.20 b	21.14 ± 3.24 b	5927.90 ± 303.74 b	3351.67 ± 667.85 b	209.71 ± 25.81 b
	Ripening	22,569.60 ± 3386.66 a	29.19 ± 4.52 a	35.25 ± 2.31 a	7341.62 ± 639.87 a	9793.08 ± 3045.70 a	511.92 ± 109.69 a
Yellow	Fruitlet	779.55 ± 74.56 ab	15.30 ± 4.40 a	3.08 ± 0.06 a	0.70 ± 0.55 c	0	2.54 ± 0.05 c
	Green	865.34 ± 36.12 abc	10.88 ± 0.71 ab	3.09 ± 0.06 a	5.88 ± 0.51 a	0	3.77 ± 0.48 b
	Veraison	565.93 ± 54.38 d	8.41 ± 0.62 c	0.65 ± 0.08 b	5.76 ± 1.67 a	0	4.88 ± 0.89 a
	Maturation	664.39 ± 34.58 bc	8.51 ± 0.62 c	0	3.66 ± 1.38 b	0.06 ± 0.11 b	3.98 ± 0.09 ab
	Ripening	705.82 ± 168.55 a	14.84 ± 4.06 a	0	2.90 ± 0.79 b	0.96 ± 0.38 a	4.59 ± 0.34 ab

Different lowercase letters represent significant differences (*p* ≤ 0.05), according to Fisher’s least significant difference (LSD) test SD (*n* = 3, 5 fruits per replicate).

**Table 2:** Validation parameters for the ultra-performance liquid chromatography (UPLC) method.

Flavonoid/Anthocyanin Component	Linearity (r^2^)	Slope (y)	Response (Sy)	Sy/y	LOD * (µg·mL^−1^)	LOQ ** (µg·mL^−1^)
Rutin	0.999303	0.2737	5.2262	19.0921	63.00	190.92
Luteolin	0.999692	0.2745	4.9727	18.1111	59.76	181.11
Quercetin	0.999667	0.2756	4.6358	16.8164	55.49	168.16
Cyanidin-3-O-glucoside chloride	0.998590	0.2767	4.3319	15.6526	51.65	156.52
Peonidin-3-O-glucoside	0.999506	0.2757	4.6096	16.7147	55.15	167.14
Pelargonidin-3-O-glucoside	0.998351	0.2754	4.7720	17.3254	57.17	173.25

* Limit of detection; ** Limit of quantification.

## Supplementary Materials

The following are available online at www.mdpi.com/2223-7747/10/11/2240/s1, Table S1: The content of apigenin and kaempferol in fruit peel of purple and yellow passion fruits during fruit development. Table S2: Sequences of primer pairs of genes responsible for flavonoids metabolism in passion fruit.
